# Raw Meat Contaminated with Cephalosporin-Resistant *Enterobacterales* as a Potential Source of Human Home Exposure to Multidrug-Resistant Bacteria

**DOI:** 10.3390/molecules27134151

**Published:** 2022-06-28

**Authors:** Bartosz Rybak, Marta Potrykus, Alina Plenis, Lidia Wolska

**Affiliations:** 1 Department of Environmental Toxicology, Faculty of Health Sciences, Medical University of Gdansk, Debowa Str. 23A, 80-204 Gdansk, Poland; bartosz.rybak@gumed.edu.pl (B.R.); marta.potrykus@gumed.edu.pl (M.P.); lidia.wolska@gumed.edu.pl (L.W.); 2 Department of Analytical Chemistry, Faculty of Pharmacy, Medical University of Gdansk, Hallera Str. 107, 80-416 Gdansk, Poland

**Keywords:** meat, ESBL *Escherichia coli*, *Serratia fonticola*, principal component analysis

## Abstract

The prevalence of cephalosporine-resistant (3GC-R) strains among United States community-related research samples ranged from 5.6 to 10.8%, while, in the European countries, it was 1.2% to 10.1%. Several studies suggest that meat of animal origin could be one of the reservoirs of 3GC-R bacteria. Here, 86 raw meat samples (turkey, pork, chicken and beef) were collected randomly and verified for the presence of 3GC-R bacteria. The 3GC-R bacteria were isolated, identified and characterized phenotypically (antibiotic resistance, motility and biofilm) and genotypically (repetitive-sequence-based rep-PCR) to elucidate any correlations with principal component analysis (PCA). From 28 3GC-R positive samples, 41 strains were isolated, from which the majority belonged to *Serratia fonticola* (39%), followed by *Escherichia coli* (19.5%), *Enterobacter cloacae* (17.1%) and *Klebsiella pneumoniae* (14.6%). The isolates of *E. coli* and *S. fonticola* presented diverse profiles in rep-PCR. Generally, 3GC-R strains were more resistant to antibiotics used in veterinary medicine than in human medicine. PCA derived from antibiotic resistance, motility and biofilm formation of *S. fonticola* and *E. coli* strains showed that resistance to beta-lactams was separated from the resistance to other antibiotic classes. Moreover, for the *S. fonticola*, *E. coli* and *En. cloacae*, the type of meat can create a specific tendency towards antibiotic resistance and phenotypic characteristics for *S. fonticola*, while these relationships were not found for other tested species.

## 1. Introduction

Antimicrobial resistance is recognized as one of the most important global health challenges because of the high risk of decreasing effective treatment of bacterial infections in both human and veterinary medicine [1,2,3], as well as increasing treatment costs [4,5]. Antibiotic-resistant infections are estimated to be responsible for approximately 2 million cases and 23,000 deaths in the United States each year [6,7]. Additionally, worldwide data indicate that 700,000 people a year die from bacterial diseases caused by the bacteria which acquired resistance genes [8,9]. Moreover, the World Health Organization (WHO) also warns that, by 2050, as many as 10 million people a year will be dying from infectious diseases caused by drug-resistant microorganisms [10,11].

The phenomenon of the antimicrobial resistance is closely correlated with the introduction of broad-spectrum cephalosporins and carbapenem antibiotics, the use of which has significantly increased the effectiveness of antimicrobial therapies in both human and veterinary medicine [12] but, at the same time, caused increasing resistance to these active substances. One of the most common resistance mechanisms in Gram-negative bacteria is enzymatic inactivation of beta-lactam antibiotics by beta-lactamases, such as extended spectrum beta-lactamases (ESBL) [13] or carbapenemases (CR) [14].

Beta-lactamases confer resistance to a variety of antibiotics, such as third- and fourth-generation cephalosporins, which are listed as “Critically Important Antimicrobials” by the WHO, as these two groups of antimicrobials provide first-line therapy for invasive Gram-negative infections in people. The encoding genes of resistance are often found in plasmids and, therefore, can be transferred horizontally, even between different species of bacteria [15]. Moreover, ESBL and CR strains often carry further resistance genes on the same plasmid, conferring resistance to several classes of antibiotics [4]. It should also be noted that ESBL and/or CR-producing *Enterobacteriaceae* were initially mainly observed in healthcare settings, including hospitals [5], but, in recent years, the number of community-acquired infections has significantly increased [16]. For example, it was noted that the prevalence of CRE strains among US community-related research samples ranged from 5.6 to 10.8% [17], while, in European countries, it ranged from 1.2% in the Czech Republic [18] to 10.1% in the Netherlands [19]. ESBL and CR genes are common in *Enterobacteriaceae*, such as *Salmonella* spp., *Klebsiella pneumoniae*, *Escherichia coli* (*E. coli*), *Enterobacter cloacae* (*En. cloacae*)*, Citrobacter* spp. and *Serratia fonticola* [8,20,21,22]. Currently, they are increasingly often found in farm animals [23], in raw meat [24], eggs [25], and food [26,27], which confirms the exchange of organisms or genes between different reservoirs [28,29]. Thus, the data suggest that farm animals and meat of animal origin are among the reservoirs of ESBL and CR bacteria [30,31,32,33,34,35,36,37,38,39,40].

The above-mentioned factors led the European Medicines Agency (EMA) to propose a new categorization of antimicrobials (EMA/Committee for Medicinal Products for Veterinary Use (CVMP)/Committee for Medicinal Product for Human Use (CHMP), 2019), which established four different categories of pharmaceuticals, from A to D. It is noteworthy that, in the category A (“Avoid”), there were included antibiotics, such as monobactams or fosfomycin, which are not authorized for use in veterinary medicine but reserved only for use in treating humans in the European Union. In the category B (“Restrict”), EMA has included antibiotics that should be restricted in animal treatment to reduce the risk for public health. In this category, quinolones, third- and fourth-generation cephalosporins and polymyxins were included [41]. These two categories of antimicrobials have also been identified as the ones most urgently in need of managing the risk of antimicrobial resistance.

On the other hand, these new categorizations and restricted use of antibiotics have not yet brought significant reduction in drug-resistant bacteria occurrence, since ESBL strains have been isolated from chicken meat in multiple places, such as Portugal, Spain, USA, the United Kingdom and Germany [42,43,44,45]. Recently, a similar distribution of the ESBL drug resistance genes has been demonstrated among raw meat isolates and the ones that were found in the samples obtained from infected humans in the Netherlands [46,47], and also in Germany [48,49]. The presence of ESBL-producing *Enterobacteriaceae* was mainly confirmed in poultry farms [50,51,52,53], but there were also reports showing their presence in pork or beef meat [54,55,56]. It should be noted that ESBL-producing *Enterobacteriaceae* can be consumed directly with raw or undercooked meat or other raw foods and cookware can act as a vector when handling contaminated meat [57]. Therefore, antimicrobial resistance may be spread by transmission of the ESBL genes to strains of human intestinal microbiota or by direct colonization of the human gut by ESBL strains [46,58]. This, in turn, may lead to an increase in infections, e.g., urinary tract infections, accounting for 75–85% of parenteral infections with pathogenic *Escherichia coli* and *Klebsiella pneumoniae* [15,33,59,60]. This is especially dangerous for patients with acquired or congenital immunosuppression and for onco-hematological patients.

So far, there are few data on the quantitative load of ESBL-producing *Enterobacteriaceae* in meat of various origins available for sale in Poland despite the fact that this country exports large amounts of raw meat to the EU and other countries in the world (1.8 million t of poultry, 744,000 t of pork and 471,000 t of beef in 2019) [61]. Moreover, the data reported by the Supreme Audit Office (NIK) show that only in five years (2011–2015) in Poland, sales of veterinary antibiotics increased by 23%; thus, Poland became one of the European countries using the largest amounts of antibiotics in animal husbandry [62,63]. Accordingly, our goal was to assess the degree of contamination with ESBL-producing Gram-negative bacteria in poultry, pork and beef meat samples that were purchased from local fresh food markets located in the territory of Gdansk (Poland). We checked whether the meat had come from local slaughterhouses or it had been supplied from other regions of Poland, and then we isolated ESBL strains which had grown from meat samples. Moreover, we conducted drug resistance tests against selected antibiotics, swimming and swarming motility and biofilm formation, as well as PCR profiles to determine a potential risk of antimicrobial resistance for consumers as the consequence of contact with the above-mentioned types of bacteria isolated in poultry, pork and beef meat. Additionally, both statistical and chemometric analyses of the obtained data based on principal component analysis (PCA) were performed to obtain a deeper understanding of relationships between the type of meat, antimicrobial resistance and results of phenotypic traits established for isolated bacterial strains.

## 2. Results

### 2.1. Most of the Third-Generation Cephalosporine-Resistant Strains Were Isolated from Turkey Meat

**Figure 1:**
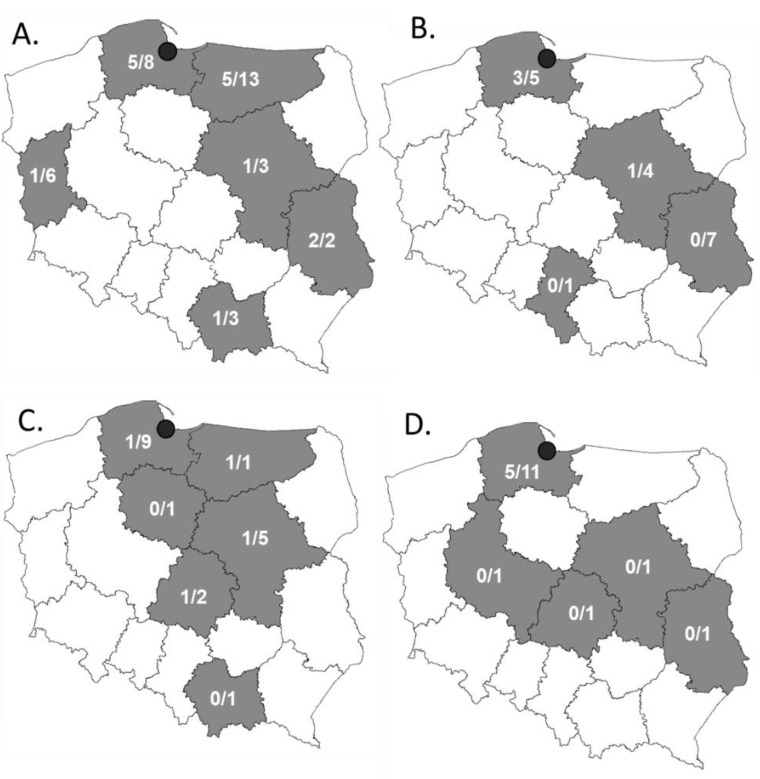
Localization of plants processing meat, from which the meat was sampled in Gdansk markets (black dot). (**A**) Turkey (*n* = 35); (**B**) chicken (*n* = 17); (**C**) pork (*n* = 19); (**D**) beef (15). In case of 11 samples of unknown producer, they were assigned to Pomorskie voivodeship.

During 2020, a total of 86 meat samples were collected in five different fresh produce markets in Gdansk (Poland) (Table 1, Figure 1, Table S1). The samples were taken from the meat randomly bought by the family of four throughout the year. The family mostly ate at home. The samples comprised mostly of the turkey meat (40.7%), followed by pork (22.1%), chicken (19.8%) and beef (17.4%). All meat samples came from animals bred on the territory of Poland. The meat was packed in two different ways, either in boxes that were air vacuumed/modified (VAC) (65%) or in polystyrene trays wrapped in transparent foil (35%). The turkey and pork meat were most frequently packed in VAC packaging (33/35 and 15/19, respectively), while chicken and pork meat were most often packed on polystyrene trays (13/17 and 11/15, respectively). The analyzed meat samples were purchased in five different markets in Gdansk, where the meat was mostly delivered (70.9%) or less frequently portioned and packed in the market (16.3%). Eleven samples (12.8%) were assigned as the market’s own brand. The place where the meat was processed could be tracked with a veterinary identification number, apart from 10 samples (mostly shops’ own brands). The meat sold in Gdansk came from 10 out of 16 voivodeships of Poland (Figure 1). The majority of the samples came from Pomeranian voivodeship (26.7%), then from Warmia-Masuria Voivodeship (16.3%), Masovian (15.1%) and Lublin (11.6%) voivodeships.

Since we wanted to mimic home conditions of handling meat, the raw meat surface was examined for bacteria contamination coming from all stages of meat processing, such as animals themselves, their breeding, as well as stages of meat processing. Among the tested samples, mostly lactose-negative bacteria were detected on MacConkey media, on average 21 ± 27 CFU/cm^2^, followed by lactose-positive bacteria at 17 ± 29 CFU/cm^2^ and mannitol-positive bacteria grown on MSA media at 10 ± 20 CFU/cm^2^ (Table 2, Table S1). The MSA mannitol-positive bacteria were mostly observed on turkey and chicken (14.9 ± 25.5 and 14.0 ± 25.5 cfu/cm^2^, respectively), while, on pork and beef, there were approximately 3–5 times less MSA mannitol-positive bacteria (5.2 ± 7.3 and 3.8 ± 6.1 cfu/cm^2^, respectively). The largest abundance of lactose-positive bacteria on MacConkey medium was observed for chicken meat samples (27.0 ± 40.1 cfu/cm^2^), while, for other meat types, it was small. In case of lactose-negative bacteria, the meat from birds (turkey and chicken) was contaminated 60% more than the meat from mammals (pork and beef) (Table 2). When looking at bacterial contamination in the context of packaging type, no significant differences could be observed (23.4 ± 27.4 and 17.5 ± 34.0 cfu/cm^2^ for vacuum and tray, respectively).

In order to isolate third-generation cephalosporine-resistant bacteria (3GC-R), the meat samples were examined with ChromAgar ESBL medium, allowing for isolation of either *E. coli* or bacteria from KESC group (*Klebsiella* spp., *Enterobacter* spp., *Serratia* spp. and *Citrobacter* spp.) that are resistant to these antibiotics. In total, the average number of *E. coli* strains grown on ChromAgar ESBL was 0.4 ± 1.2 cfu/cm^2^, while the average number of KESC grown on the same medium was 5.6 ± 16.9 cfu/cm^2^. From 86 meat samples that were examined, 28 (32.5%) were positive for 3GC-R strains’ presence. The majority of the 3GC-R strains were isolated from turkey meat (15/35), which was contaminated with such strains in 43% cases. Other meat types were less contaminated with 3GC-R strains (5/15, 4/17 and 4/19 for beef, chicken and pork, respectively), but still as many as 33% to 21% of the meat samples were contaminated.

From 28 3GC-R-positive samples of raw meat, 41 strains were isolated. The strains were identified with MALDI-TOF MS analysis. The isolated strains belonged to six species and four genera. The majority of the 3GC-R strains belonged to *Serratia fonticola* (39%), followed by *Escherichia coli* (19.5%), *Enterobacter cloacae* (17.1%), *Klebsiella pneumoniae* (14.6%), *Serratia liquefaciens* (7.3%) and *Serratia marcescens* (7.3%) (Table 3). The majority of *S. fonticola* isolates were found on turkey and chicken meat (six and four isolates, respectively), while most of the *E. coli* and *K. pneumoniae* strains were isolated from turkey meat (seven and five strains, respectively). More *E. coli* and *S. fonticola* strains were isolated from meat packed with VAC system than that on trays (nine and seven strains, respectively). For 63% of isolated strains, the phenotypic confirmation of ESBL mechanism was positive in the double-disk synergy (DDS) test, while, for 37% (15 strains), these mechanisms could not be verified. Among *S. fonticola* and *E. coli* strains, 12/16 and 6/8 strains were ESBL-positive, respectively. For *S. liquefaciens*, two out of three strains scored positive in DDS test, while, for *En. cloacae*, two out of six were positive. 

The antibiotics that were the most active against the tested strains were beta-lactams from the carbapenem group, as only for imipenem and meropenem the tested strains were not fully sensitive (7.3% and 12.2%, respectively).

**Figure 2:**
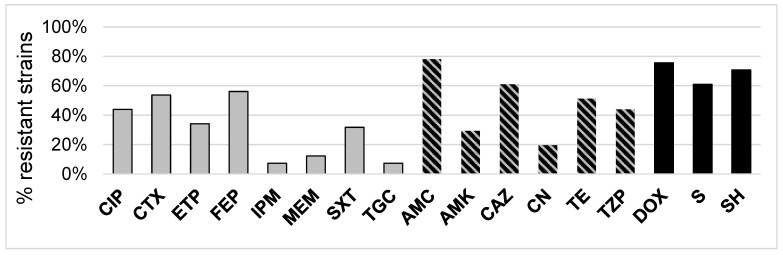
The antibiotic resistance of the tested cephalosporine-resistant strains isolated from meat samples (*n* = 41). All the strains were resistant to ampicillin (AMP) and cefuroxime (CXM); thus, these antibiotics were excluded from the graph. In black: antibiotics used only in veterinary therapies; in grey: antibiotics used only in human therapies; black-grey stripes: antibiotics used both in veterinary and human therapies; AMC—amoxicillin/clavulanic acid; TZP—piperacillin/tazobactam; CTX—cefotaxime; CAZ—ceftazidime; FEP—cefepime; IPM—imipenem; MEM—meropenem; ETP—ertapenem; AMK—amikacin; CN—gentamycin; CIP—ciprofloxacin; SXT—co-trimoxazoles; TGC—tigecycline; TE—tetracycline; DOX—doxycycline; S—streptomycin; SH—spectinomycin.

The antibiotics from the aminoglycosides group were less effective against the tested bacterial strains, as 19.5% strains were resistant to gentamycin and almost one third were resistant to amikacin (AMK). The least effective were beta-lactam antibiotics from penicylin group with beta-lactam inhibitors (amoxicillin/clavulanic acid (AMC) and piperacillin/tazobactam (TZP)), to which 78% and 43.9% of strains were resistant, respectively (Figure 2). Surprisingly, not all strains that were isolated as 3CG-R were resistant to all cephalosporins of third and fourth generation in the in vitro tests. Interestingly, among *S. fonticola* strains, one strain was resistant to imipenem (IPM), two strains were resistant to meropenem (MEM) and as many as five were resistant to ertapenem (ETP). Among *En. cloacae* and *K. pneumoniae* isolates, there were single strains that were also less sensitive to ETP. This unexpected resistance to the mentioned antibiotics needs further verification. 

When taking into account antibiotics used for human and veterinary medicine, the overall resistance to veterinary antibiotics was much higher than resistance to antibiotics used for human treatment, apart from aminoglycosides (AMK—29% and gentamycin (CN) 20%). The tested strains were fully resistant to ampicillin and cefuroxime. Apart from these two, the largest number of resistant strains observed was for AMC (78%), doxycycline (DOX) (76%), spectinomycin (SH) (71%), ceftazidime (CAZ) (61%) and tetracycline (TE) (51%) (Figure 2, Table S3). 

### 2.2. 3CG-R Strains Isolated from Meat Express Diversity in Phenotypic Traits

**Figure 3:**
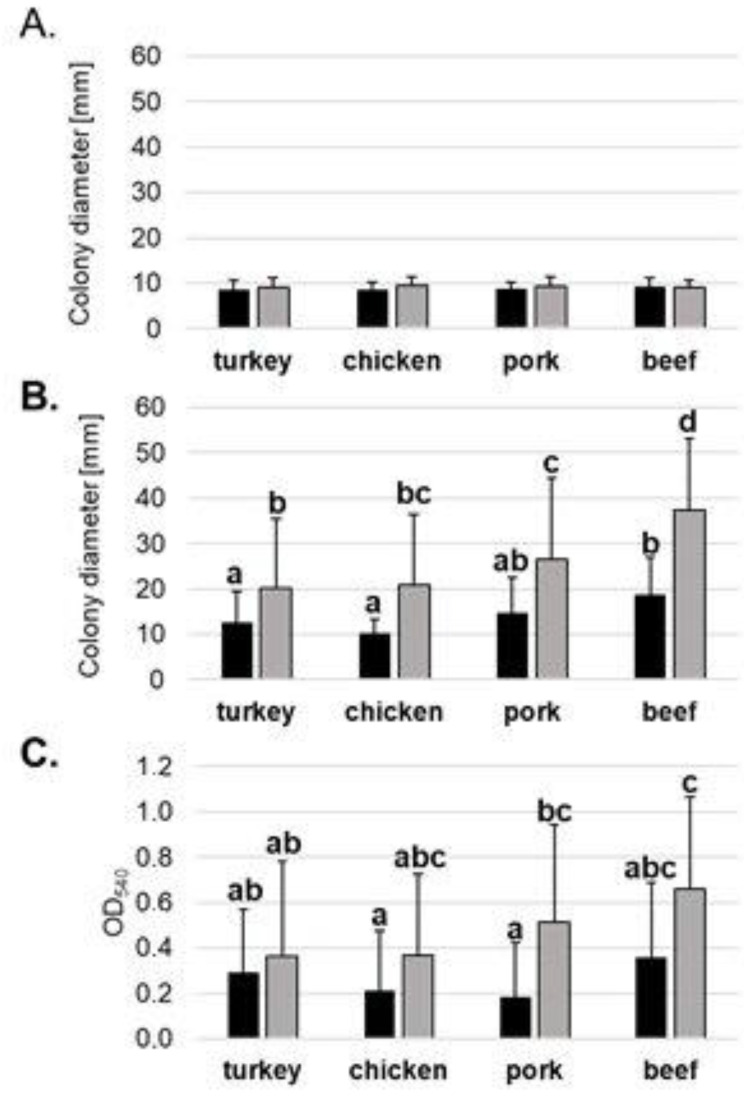
Swarming (**A**), swimming (**B**) and biofilm formation (**C**) ability at room temperature (black) and at 37 °C (grey) of tested cephalosporin-resistant strains isolated from meat samples. The results are presented as an average for isolated bacteria from each meat type (turkey *n* = 23; chicken *n* = 6; pork *n* = 7; beef *n* = 6). The experiment was performed twice with at least three replicates. Statistically different results are marked with different letters as tested with ANOVA followed by Tuckey post hoc test at *p* < 0.05.

The 3CG-R strains that were isolated from four types of meat samples (turkey, chicken, pork and beef) were subjected to phenotypic analysis to determine differences in their ability for survival and colonization. The strains were tested for their ability to swim and swarm on LA plates with different amounts of agar, and their biofilm formation capability was verified at two different temperatures. The results obtained for separate strains were then used to compare the phenotypic traits for the separate species as well as to seek any differences regarding the type of meat the strains were isolated from (Figure 3 and Figure S1). As regards the species, there could be no influence observed of the temperature of incubation on the swarming motility of the strains. Moreover, swarming motility was similar for each tested species, as well as for the two reference strains used in the study (*S. marcescens* C19 and *E. coli* C49). For swimming motility, a noticeable increase in the colony diameter could be noticed for all tested species but *K. pneumoniae* and *E. coli* C49 reference strain. In the case of *S. fonticola*, *S. liquefaciens*, *En. cloacae* and *E. coli*, the increase in swimming motility at 37 °C was statistically significant. As to biofilm formation, a slight increase in biofilm formation ability at 37 °C could be observed; however, it was significant only in the case of *K. pneumoniae* (Figure S1). As to the strains’ phenotypic traits in the view of meat type, swarming ability did not depend on either the temperatures of incubation or different meat types. As regards swimming ability at room temperature, the strains isolated from turkey and chicken were least motile, while the strains isolated from beef were significantly more motile than strains isolated from turkey and chicken. Similarly, the motility of all the strains increased with temperature being significantly higher for strains isolated from all meat types. At 37 °C, the strains isolated from beef were significantly more motile than strains isolated from turkey, chicken and pork. Biofilm formation was increased for strains isolated from each meat type; however, this increase was significant only for pork and beef isolates. Similar to swimming ability, the biofilm formation capability of the strains isolated from beef was the highest among the strains tested (Figure 3).

### 2.3. Repetitive Sequence Profiles Are Diverse among E. coli and S. fonticola 3CG-R Strains

**Figure 4:**
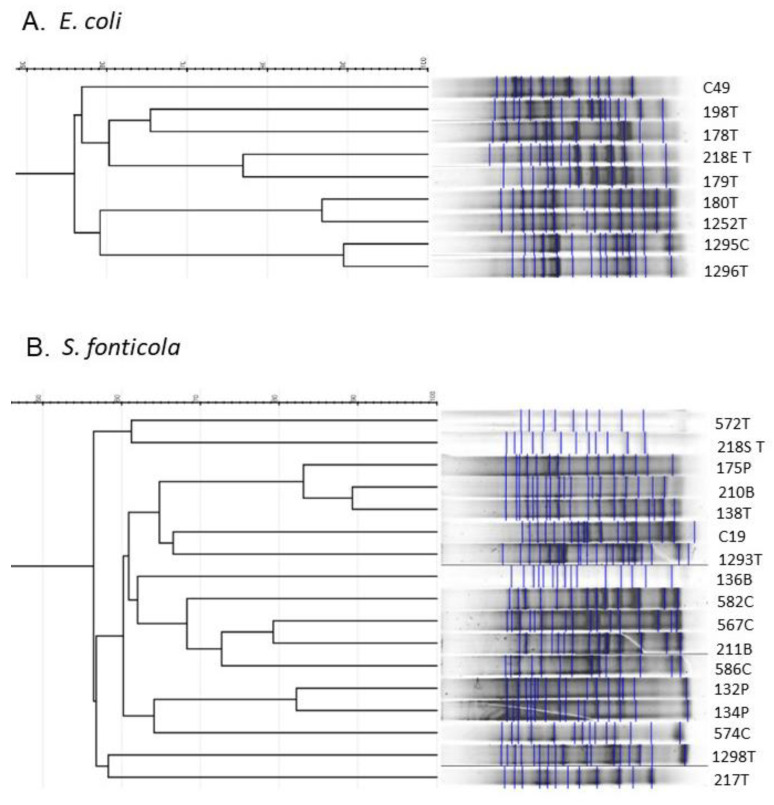
Fingerprinting profiles of *E. coli* (**A**) and *S. fonticola* (**B**) cephalosporin-resistant strains isolated from meat samples. The profiles were obtained with ERIC rep-PCR primers and analyzed with GelJ software using UPGMA and band similarity index set to 1.0. T—turkey, C—chicken, P—pork, B—beef, C49—*E. coli* ATCC 25922; C19—*S. marcescens* KPD102-BA.

When looking at repetitive sequence profiles obtained with rep-PCR with ERIC primers for the most abundant species, *S. fonticola* (*n* = 16) and *E. coli* (*n* = 8), large variability in the profiles could be visible (Figure 4). All *E. coli* 3CG-R strains were isolated from poultry (mostly from turkey). A total of seven different band patterns could be distinguished in the analysis, with *E. coli* C49 reference strain being diverse from each of the tested isolates. A few identical patterns could be observed for this species (strains 1295C and 1296T; 180T and 1252T). It is noteworthy that all identical pairs were comprised of the isolates coming from different voivodeships. Moreover, *E. coli* 1295C and 1296T were isolated from two different meat types (chicken and turkey, respectively). The *S. fonticola* isolates represented more diverse repetitive sequence profiles than *E. coli* isolates, as, for 16 strains taken for the analysis, as many as 12 different profiles could be distinguished. Similar to *E. coli* isolates, in three cases, the profiles were almost identical to each other (strains 175P, 210B, 138T and 132P, 134P and 211B, 582C). One pair of *S. fonticola* strains (132P and 134P) with identical profiles was isolated from the same type of meat (pork), and the meat came from the same voivodeship. In the case of *S. fonticola* 175P, 210B and 138T, despite having the same profiles, the strains were isolated from diverse meat types (pork, beef and turkey) produced in different voivodeships, similarly to *S. fonticola* 211B and 582C strains. 

### 2.4. Antibiotic Resistance Patterns May Be Explained by Meat Type for S. fonticola and En. cloacae 

**Figure 5:**
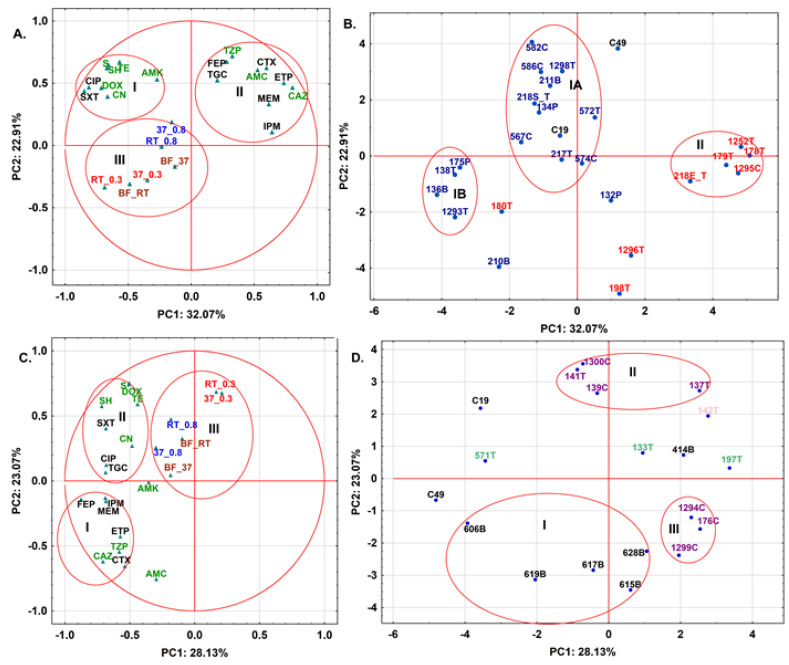
The PCA results for variables (**A**,**C**) and objects (OTUs) (**B**,**D**) based on data for antibiotic resistance (excluding ampicillin (AMP) and cefuroxime (CXM)) and phenotypic tests (swimming, swarming and biofilm formation) for tested cephalosporine-resistant strains (*E. coli* and *S. fonticola* (**A**,**B**) and *K. pneumoniae, En. cloacae, S. liquefaciens and S. marcescens* (**C**,**D**)) isolated from meat samples. In (**A**,**C**): veterinary antibiotics (green), human antibiotics (black); AMC—amoxicillin/clavulanic acid; TZP—piperacillin/tazobactam; CTX—cefotaxime; CAZ—ceftazidime; FEP—cefepime; IPM—imipenem; MEM—meropenem; ETP—ertapenem; AMK—amikacin; CN—gentamycin; CIP—ciprofloxacin; SXT—co-trimksazol; TGC—tigecycline; TE—tetracycline; DOX—doxycycline; S—streptomycin; SH—spectomycin; RT—room temperature; 37C—37 °C; 0.3—swimming motility; 0.8—swarming motility; BF—biofilm formation. In (**B**,**D**): T—turkey; B—beef; P—pork; C—chicken. *E. coli* (red); *S. fonticola* (blue); *K. pneumoniae* (black); *En. cloacae* (purple); *S. liquefaciens* (green); *S. marcescens* (orange); C49—*E. coli* ATCC 25922; C19—S. *marcescens* KPD102-BA were used as reference strains.

Next, PCA derived from the autoscaled data of antibiotic resistance of tested strains (excluding ampicillin (AMP) and cefuroxime (CXM)) and phenotypic tests (swimming, swarming and biofilm formation) for tested 3CG-R strains isolated from meat samples was performed. For a more detailed interpretation of the obtained results for the studied samples, a statistical technique, namely PCA, was applied. The PCA belongs to a group of factor analysis and offers graphic data visualization of the relationships between the variables or objects without losing any significant information. This approach is based on the transformation of the original measured variables into new uncorrelated variables called principal components (PC), where each PC is a linear combination of the original responses and is orthogonal to each other [64]. Thus, the first PC (PC1) explains most of the variation in the data, whereas PC2 covers much of the remaining variation, and so on. That means that a few PCs explain the variation of a large number of original responses. Thus, a two-dimensional or three-dimensional projection of samples which are based on the PC axes allows the relationships between objects to be shown, looking for groups and trends, and sorting out outliers. The PCA results calculated for the meat samples with isolated *E. coli* (8) and *S. fonticola* (16) are shown in Figure 5A (variables) and 5B (objects, OUTs), while the ones with *K. pneumoniae* (6), *En. cloacae* (7), *S. liquefaciens* (3) and *S. marcescens* (1) are presented in Figure 5C (variables) and 5D (objects, OUTs), respectively. In both PCA calculations, the autoscaled data obtained for *E. coli* C49 and *S. marcescens* C19 used as the references were also included. In the case of PCA calculation based on the matrix data obtained for the meat samples with isolated *S. fonticola* and *E. coli* strains (26 objects x 23 variables), the first two PCs explained 54.97% of the data variability (Figure 5A,B). Moreover, the variances in the variables describing resistance to sulfamethoxazole/trimethoprim (SXT) and ciprofloxacin (CIP) of *E. coli* and *S. fonticola* isolated from the tested meat samples and then to CAZ and ETP were mainly explored by the PC1, whereas the variabilities in resistance parameters to TZP and cefepime (FEP), as well as TE and SH, were mainly explained by the PC2. Thus, the variability in the phenotypic analysis for the tested strains was not explored by the two first PCs. According to the loading PCA plot (Figure 5A), the parameters describing the resistance to SXT, SH and TE were located on the left part of the upper side of the graph together with those calculated to aminoglycosides (AMK, CN and streptomycin (S)), DOX and SH (cluster I). In the case of CAZ, ETP, TZP and FEP, they were positioned with the antibiotics belonging to penicillin (AMC), cephalosporin (cefotaxime (CTX)) and carbapenem group (IMP, MEM) (cluster II) on the right side of the plot, respectively. Only tigecycline (TGC) and AMK were found in opposite clusters taking into account their chemical structures. This indicates that PCA distinguished tested antibiotic resistance of *S. fonticola* and *E. coli* strains according to beta-lactamase activity. It is also interesting that, when the application in human and veterinary medicine was taken into account, six out of eight antibiotics used for human treatment were located in cluster I, while seven out of nine of veterinary drugs were positioned in cluster II, respectively. Thus, PCA results also confirmed that the way an antibiotic is used can be considered as a deciding factor regarding creation of specific antibiotic resistance in the strains. The phenotypic results of biofilm formation (BF_RT and BF_37), swimming (RT_0.3 and 37_0.3) and swarming (37_0.8 and RT_0.8) were located in cluster III. These parameters gave comparable characteristics of the tested strains but these results were different in respect to resistance established for antibiotics (Figure 3 and Figure S1). Thus, for *E. coli*, a pair of strains isolated from the same meat type (turkey) falls close to each other in PCA analysis taking into account antibiotic resistance and phenotypic traits of the strains. On the other hand, the third pair (1295C and 1296T) that was isolated from chicken and turkey, despite having the same genotypic profiles, represents diverse phenotypic traits in PCA analysis. In case of *S. fonticola*, the strains with similar band patterns presented diverse tendencies in PCA analysis of phenotypic traits. The *S. fonticola* 132P and 134P, despite both being isolated from pork, express different behavior in phenotypic tests, while *S. fonticola* 582C and 211B isolated from chicken and beef express very similar phenotypic features in the tested conditions. As was mentioned above, the variability in these parameters was not explained by the first two PCs. This fact explains why the localization of the tested meat samples with isolated *S. fonticola* and *E. coli* strains in the score PCA plot (Figure 5B) was not consistent to the observations based on phenotypic analysis. 

Thus, the score plot for the object (OTUs) based on the above-mentioned data set showed that most meat samples were located according to the species of the tested strains isolated from these samples (Figure 5B). So, eight poultry, one bovine and one pork meat *S. fonticola* isolates were positioned in cluster I A, together with *S. marcescens* C19, whereas four other strains (138T, 1293T, 136B and 175P) were found in cluster I B. The small distance between these clusters indicated that slight differences in the antibiotic resistance and maybe phenotypic profiles were found between these samples. Only the strains 210B and 132P isolated from bovine and pork meat samples, respectively, were located as outliers on the PCA plot. Thus, these data suggest that the samples with *S. fonticola* isolated from turkey meat had more comparable profiles in respect to those determined for beef and pork meat. Thus, the type of meat can offer a specific tendency towards antibiotics resistance and maybe phenotypic properties for *S. fonticola*. In the case of *E. coli* isolates, five out of eight poultry meat isolates were positioned in cluster II, while 1296T and 198T were located in the bottom side of the PCA plot. Due to the fact that only poultry meat samples with *E. coli* were tested (seven from turkey and one from chicken), it was impossible to verify any relationship between type of meat and antibiotic resistance or phenotypic traits. It should be also noticed that the behavior of *E. coli* C49 was different in the tested experimental conditions, which was found as an outlier in the upper part of the PCA plot. When PCA was based on the matrix data (19 objects × 23 variables) calculated for the meat samples with four other species, the first two PCs allowed 51.19% of the total variance to be explained (Figure 5C,D, respectively). In this PCA calculation, the variances of other antibiotic resistance were mainly explored by the first PCs in respect to *S. fonticola* and *E. coli* (Figure 5A,B). Thus, the resistance to FEP, CAZ, IPM and MEM for *K. pneumoniae*, *En. cloacae*, *S. liquefaciens* and *S. marcescens* strains isolated from the tested meat samples was mainly described by PC1, while PC2 explored the variability in resistance to AMC, CTX, DOX and S, respectively. The loading PCA plot illustrated in Figure 5C showed that these parameters, except for AMC, were located in cluster I (FEP, IPM, MEM, CAZ and CTX) and cluster II (S and DOX), respectively. It should be noticed that the parameters describing the resistance to ETP and TZP were also located in cluster I, whereas resistance to AMC was found as an outlier in a small distance to cluster I. Thus, four species of isolated strains from the meat samples possessed comparable profiles of resistance to beta-lactam antibiotics and the drugs belonging to other antibiotic classes, which were included by PCA into cluster II. Only the variable calculated for resistance to AMK was located as an outlier between clusters I, II and III, respectively. It can also be observed that most antibiotics used in veterinary treatment (six) were positioned by PCA in cluster I, when TGC, CIP and SXT were found in cluster II together with five other drugs used in human medicine. On the other hand, the variables describing the resistance to CAZ and AMC were located at a long distance to AMK and the drugs positioned in cluster II. Thus, the positions of human antibiotics in the loadings PCA plot were more different than those found for *S. fonticola* and *E. coli* strains (Figure 5B). It can also be noticed that swimming ability (RT_0.3 and 37_0.3) provided slightly different data about the strains than swarming and biofilm formation profiles for *S. fonticola* and *E. coli* strains (Figure 5A). However, in PCA calculation based on the data set calculated for the other four species, the first two PCs did not explain the variabilities in these parameters for the tested strains. This can explain the lack of correlation between the phenotypic characteristics of the tested bacterial isolates and their localizations in the score PCA plot illustrated in Figure 5D. 

Therefore, the graphical data shown in Figure 5D confirmed that four *K. pneumoniae* turkey meat isolates (606T, 619T, 628T and 615T) and one isolated from pork (617P) were included in cluster I positioned in the bottom side of the graph, while *K. pneumoniae* 414T was found at a small distance to *S. liquefaciens* (133P and 197T) and *S. marcescens* (142B) on the right side of the graph. 571T also containing *S. liquifaciens* was positioned as an outlier between *E. coli* C49 and *S. marcescens* C19 on the left of the PCA plot. This suggested that various antibiotic resistance profiles were calculated for the used reference and the above-mentioned strains. Moreover, there were no relationships between the type of the tested meat samples and the antibiotic resistance for *K. pneumoniae*, *S. liquefaciens* and *S. marcescens* strains. On the other hand, these profiles were also different in respect to the ones calculated for *En. cloacae* strains. It is interesting that, for this species, only beef and pork meat isolates (1300B and 141B, and 139P and 137P, respectively) were included in cluster II, while the strains isolated from turkey and chicken (175T, 1294T and 1200TC) were found in cluster III. Thus, the type of meat can designate specific antibiotic resistance profiles. 

Summarizing, PCA derived from antibiotic resistance of the tested strains (excluding AMP and CXM) and phenotypic trials (swimming, swarming and biofilm formation) for the tested cephalosporine-resistant strains isolated from meat samples showed that parameters describing resistance to beta-lactam antibiotics were separated from the resistance to other classes of tested antibiotics and phenotypic traits for both *S. fonticola* and *E. coli* and four other species. Most antibiotic resistances calculated for human and veterinary drugs were assigned to specific clusters for *S. fonticola* and *E. coli* but this observation was not found for the antibiotics used in human medicine when four other tested species isolated from the meat samples were used in PCA calculation. In both loadings PCA plots, biofilm formation, swimming and swarming were found within the same cluster, which can suggest that these parameters gave comparable differentiation of the tested strains, but this description was different than the one observed for antibiotic resistance. However, in both PCA calculations, the first two PCs did not explain the variabilities of these parameters for the tested strains. In the case of score PCA plots established for the *S. fonticola* and *E. coli*, the type of meat can offer a specific tendency towards antibiotic resistance and maybe phenotypic properties for *S. fonticola*. The same effect was found for *En. cloacae* in the calculation based on the data set established for the other four species, while these relationships were not confirmed for other tested species.

## 3. Discussion

The average consumption of meat on the territory of the European Union equals 70.3 kg/person/year, which is about 40% more than in China (49.3 kg/person/year) and approximately 30% less than in the USA (100.7 kg/person/year) [65]. Moreover, the consumption of different meat types (poultry, pork and beef) also shows diverse patterns between the mentioned regions, where, for the EU and USA, poultry is the largest share (47 and 51%, respectively), while, for China, the pork meat accounts for the most consumed meat type (63%) [65]. The consumption of beef is declining in the EU, while the consumption of poultry is constantly rising when looking back from 2010 to 2020 [65].

In Poland, the average meat consumption accounts for 61 kg/person/year [66], which is about 15% less than the EU average. However, Poland is one of the largest meat producers among EU countries, accounting for 20% of poultry production (first place) and 9% for pork production (fifth place) in the EU in 2020 [67,68]. What is more, in Poland, penicillin sales for food-producing animals reached 54.1 mg/PCU (mg per population correction unit) in 2017, being the fourth largest after Cyprus, Italy and Spain among European countries [69].

In our study, throughout the year, there were collected 86 meat samples, accounting for about 61% of an average person’s meat consumption in the EU. However, the proportions of the poultry/pork/beef were a bit different than in the EU (60.5% poultry, 22.1% pork and 17.4% beef). On average, 32.5% of the samples were positive for 3GC-R strains’ presence, with different prevalence when taking into account the meat type. Similarly, 3GC-R *E. coli* have been found in meat from all animal species across Europe [70], despite the fact that the use of third- and fourth-generation cephalosporins is not authorized for poultry in the European Union (2015/C 299/04). Noteworthy, human infections with 3GC-R *E. coli* and other *Enterobacterales* are associated with substantial morbidity, mortality and increased costs compared to infections caused by pathogens susceptible to antibiotics [71,72]. Originally, 3GC-R and CR *Enterobacterales* infections were mainly a hospital-related problem with bacteria acquisition in hospitals or related to healthcare contact. This has changed in the past two decades, with people that have had no healthcare contact also being rectal carriers of 3GC-R and CR strains [73]. Many recommendations of international medical associations still include 3GC as the first-line drugs in the empirical treatment of community-acquired infections, especially in patients not previously hospitalized. The presence of 3GC-R strains in raw meat, which is a popular food product, poses a risk of acquiring colonization and/or infection with 3GC-R strains and, thus, the ineffectiveness of 3GC empirical therapy. Empirical therapy for community-acquired bacterial infections, such as urinary tract infection or Gram-negative sepsis, is the first line of saving the patient’s life. The waiting time for a microbiological test, even with the use of the latest diagnostic methods, will extend the time of using an adequate antimicrobial drug. At a time when the number of patients with acquired or congenital immune disorders is constantly increasing, the uncertainty of the use of drugs considered to be the most effective way to fight community-acquired infections limits the possibilities of effective treatment of this type of infection.

The most abundant in 3GC-R strain presence in our study was turkey meat (43%), which was similar to the prevalence found in Germany (40%) [74]. When taking into account chicken meat, in Poland, the prevalence of 3GC-R strains was 33%, while, in other countries, it was usually higher. For example, in Germany, Bangladesh and South Korea, 3GC-R strains on chicken meat were found in as many as 70–74% of the samples [74,75,76], respectively]. A lower amount of 3GC-R producers were found in Pakistan (39%), Spain (43%), the Netherlands (54%) and Singapore (51%), still being at least 6% higher than in the presented study [24,77,78,79]. The highest amount of 3GC-R strains was found in France (92%), for chicken breast samples [50]. As regards different European countries, the proportion of broiler meat contaminated with 3GC-R strains is, on average, larger than in the single studies, as shown by EFSA report expanding from 0.4% for Norway up to 78% for Spain [80].

As for pork meat, the prevalence of 3GC-R strains in Polish meat samples (24%) was similar to that found in other countries (Singapore (27%), South Korea (30%) and Germany (12%)) [24,73,75]. Interestingly, the prevalence of the 3GC-R strains was the lowest in beef meat (21%); however, this value was still at least three times higher than in other countries (Germany 4.2%, South Korea 0% and Singapore 7%) [24,74,76]. Although beef meat seems to be relatively less contaminated with 3GC-R strains than other meat types, the risk for human exposure to these strains is the highest, accounting for as much as 61% of total exposure in the Netherlands [81], despite the fact that the highest contamination of *E. coli* 3GC-R strains was observed for chicken meat.

However, most of the above-mentioned abundance of 3GC-R strains in different meat types reflected only *E. coli* species. In our study, we determined the presence of 3GC-R strains belonging to six different species in meat samples. Similarly, in Spain, as many as 10 species were identified in meat samples and, in Turkey, four 3GC-R species were found in chicken meat [78,82]. In the presented study, the most prevalent were *S. fonticola* (39% of isolates) and *E. coli* (20%) species, followed by *En. cloacae* (17%) and *K. pneumoniae* (15%). In Spanish meat samples, all four mentioned species were also identified, with the largest prevalence of *E. coli* (71%), followed by *S. fonticola* (14%) [78]. Similarly, Özpınar et al. [82] found *E. coli* (37%) to be the most prevalent species in examined chicken meat samples, followed by *En. cloacae* and *K. pneumoniae* (both 2.5%). Of note, we have not only identified the 3GC-R strains to the species level, but also performed a genotypic profiling of the most abundant ones (*E. coli* and *S. fonticola*). Similar to other studies, the obtained profiles were diverse [31,50,76]. Interestingly, in a few cases, we have found strains with identical profiles, both isolated from the same type of meat or from different types and of different origin. Kim et al. [76] have also found two *E. coli* strains with 99% identical profiles isolated from meat samples in Singapore coming from the USA and Brazil.

Moreover, the isolated 3GC-R strains were more resistant to antibiotics used in veterinary medicine than those in human medicine and differences could be noticed between resistance patterns of strains isolated from different meat types (see: PCA analysis). This phenomenon clearly suggests that the strains express a “meat type” resistance phenotype which would be likely to develop, as, for different animals, different antibiotics are more frequently used for treatment. In Poland, the usage of antibiotics in animal breeding has grown recently; however, in numerous countries, the usage of antibiotics in meat production was decreased in the past decade [50,79]. In such a case, it would be expected that the prevalence of 3GC-R bacteria would also decrease in raw meat, which was shown by Huizinga et al. [79] for chicken meat, where a drop from 68.3% of positive samples in 2014 to 44.6% in 2015 was observed. In contrast, Casella et al. [50] have shown that, at least in France, for the chicken breast, no decrease in 3GC-R strain prevalence was observed.

The rationale of 3GC-R strain transmission between people (farm workers, local residents and, in particular, consumers) and animals, slaughter houses and meat processing plants are not fully explored. The publications mainly compare the strains’ genotypes or the plasmids carried by the strains, which, in terms of horizontal gene transfer, does not always clearly indicate an epidemiological pathway [83]. In Denmark, the cross-transmission of resistant bacteria between broiler breeding and workers has been proven [81]. Consequently, it could be expected that a fraction of these bacteria are transferred from raw chicken meat to other foods through cross-contamination and recontamination processes—and, ultimately, to the consumer through the intake of these contaminated foods. Bacteria are able to attach to surfaces and form biofilms on them, which was also shown in this study. Interestingly, the isolates coming from turkey meat, which was the most contaminated with 3GC-R strains, have shown a relatively high biofilm formation ability at room temperature, unlike strains isolated from other meat types, for which a large difference between incubation temperatures was observed. Biofilm-embedded bacteria present increased resistance to antimicrobial treatments. Detachment of biofilm cells may lead to the cross-contamination of the products [84]. The problem of biofilm production and high levels of swimming and swarming abilities of strains derived from food is also a risk factor for cross-contamination during raw meat processing at home, where there may be cross-transmission of food-borne or multi-drug-resistant strains from food to the work area in the kitchen. High biofilm formation in food-borne strains may favor cross-transmission in home kitchens and small local restaurants. Indeed, 18% of total exposure was due to cross-contamination via meat handling at home [81]. The meat samples tested came from one family, where the rules of preventing cross-contamination while handling meat were carefully followed. In the gastrointestinal tract of the family members (from which one suffers from inherited immunodeficiencies), no 3GC-R strains were found at the end of the project (data not shown).

In the prevention of human infections, the source of the infection is as important as the route of infection in order to be eliminated or reduced. An important factor in preventing the acquisition, colonization and possibly infection with 3GC-R strains is to obey basic hygienic rules when handling raw meat and meat products during their production and preparation of meals at home. Eating raw or uncooked meat carries the risk of infection not only with 3GC-R strains, but also with other common pathogens of the gastrointestinal tract [85]. More awareness should be raised among consumers and meat producers in order to decrease the risk of becoming infected with 3GC-R strains.

## 4. Materials and Methods

### 4.1. Fresh Produce Samples

A total of 86 fresh retail meat samples (35 turkey, 17 chicken, 19 pork and 15 beef) were collected in 5 different semi and large size hypermarkets in the city of Gdansk (Pomerania, Poland) from 7 January 2020 to 21 July 2021. The examined samples reflect about 70% of a typical annual meat intake of an average person in Poland (annual intake = 61kg/person/year; [66]). The tested meat samples were purchased in generally accessible stores for retail customers; there was no co-operation with sellers, distributors or meat producers. The meat bought in stores was previously packed in 2 VAC systems or on foamed polystyrene trays wrapped with foil. Of the 86 samples tested, 56 (65%) were packed in vacuum packaging or modified atmosphere packaging, while 30 (35%) samples were bought on foamed polystyrene trays wrapped with transparent foil.

### 4.2. Isolation of Cephalosporin-Resistant Bacterial Strains

The meat samples were processed within 24 h after buying. The piece of meat was neither washed nor sterilized before use so as to mimic conditions of meat processing at home. The meat was imprinted on several media, such as MacConkey (Graso Biotech, Jabłowo, Poland) for determining the presence of lactose-negative and lactose-positive *Enterobacterales*; Mannitol Salt Agar (Graso Biotech) for *Staphylococci* growth, MRSA ChromAgar (Graso Biotech) for methicillin-resistant *Staphylococci* and ESBL ChromAgar (Graso Biotech) for the presence 3GC-R *E. coli* and bacteria constituting the KESC group (*Klebsiella*, *Enterobacter*, *Serratia*, and *Citrobacter*). The imprints were then incubated for 24–48 h at 37 °C. The characteristic colonies grown on each media were counted and CFU/cm^2^ of the meat surface was calculated. Additionally, for ESBL ChromAgar positive plates, 1–4 characteristic colonies were transferred to a fresh medium and grown until pure. Pure colonies were identified with the use of MALDI-TOF MS (Matrix-Assisted Laser Desorption/Ionization with Time of Flight Mass Spectrometry) (MALDI Biotyper; Bruker Daltonics, Billerica, MA, USA) according to the manufacturer’s procedure. Strains identified with MALDI-TOF MS were also stored in the laboratory collection at −60 °C with 20% glycerol (Epoch, Poland) [8].

### 4.3. Antimicrobial Susceptibility Testing

The double-disk synergy (DDS) assay was carried out to identify ESBL-producing strains [86]. Subsequently, *E. coli* and KESC group isolates were tested using a Kirby–Bauer disk diffusion assay for susceptibility to the following 19 antimicrobial agents according to the European Committee for Antimicrobial Susceptibility Testing (EUCAST) v.10.0 (2020) guidelines and according to Clinical and Laboratory Standard Institute CLSI VET01S ED5:2020 guidelines for veterinary chemotherapeutics: ampicillin (AMP) (10 µg), amoxicillin/clavulanic acid (AMC) (10/30 µg), piperacillin/tazobactam (TZP) (30/6 µg), cefuroxime (CXM) (30 µg), ceftazidime (CAZ) (10 µg), cefepime (FEP) (30 µg), cefotaxime (CTX) (5 µg), ciprofloxacin (CIP) (5 µg), gentamycin (CN) (10 µg), amikacin (AMK) (10 µg), imipenem (IPM) (10 µg), meropenem (MEM) (10 µg), ertapenem (ETP) (10 µg), tigecycline (TGC) (15 µg), sulfamethoxazole/trimethoprim (SXT) (25/25 µg), doxycycline (DOX) (30 µg), tetracycline (TE) (30 µg), streptomycin (S) (25 µg), and spectinomycin (SH) (25 µg) (Oxoid). *E. coli* ATCC 25922 (C49) was used as a reference strain.

### 4.4. Motility and Biofilm Formation

The motility (swimming and swarming) as well as biofilm formation assays were performed as in Rybak et al. [8]. Briefly, the bacteria were grown overnight at 37 °C, then the bacterial suspensions were prepared (0.5 MacFarland units). Each suspension in the volume of 10 µL was put on LA medium with a decreased amount of agar—0.3% for swimming and 0.8% for swarming motility and incubated for 24 h at 37 °C. For biofilm formation, the bacterial suspensions were 10× diluted in the ½ LB medium (decreased amount of yeast extract and peptone) and grown in 24-well Nunclon Delta Surface (Thermo Scientific) plates for 48 h at 37 °C without agitation. After incubation, the cultures were discarded and formed biofilm stained with crystal violet for 20 min without agitation. Then, the wells were washed 4 times with 900 µL of distilled water. Afterwards, 900 µL of ethanol (96%, Sigma Aldrich, Darmstadt, Germany) was added into each well and the OD_540_ was measured. The tests were performed twice (motility) and three times (biofilm formation) with at least 2 replicates for each strain. As the reference for the above-mentioned assays, *E. coli* ATCC25922 (C49) and *Serratia marcescens* KPD102-BA (C19) were used.

### 4.5. DNA Fingerprinting

For bacterial strains that belonged to *S. fonticola* and *E. coli* species, which were the most abundant, genomic DNA was isolated with Genomic Mini AX Bacteria Kit (A&A Biotechnology, Gdynia, Poland). Then, the DNA concentration was measured (Epoch Microplate Spectrophotometer; BioTek Instruments, Agilent, Santa Clara, CA, USA) and the genomic DNA was analyzed with repetitive-sequence-based rep-PCR with ERIC primers [87]. After performing PCR reactions, 5 µL of the PCR products was resolved in 0.8% agarose gels (0.5xTBE) at 50 V for 2.5 h. After obtaining a band pattern for each bacterial strain, they were analyzed with GelJ [88]. Similarity trees were composed with the unweighted pair group method with arithmetic mean (UPGMA) with band difference 1.0. The similarity cut-off value for the pair of strains treated as having the same profile was set to >0.5. *E. coli* strains C49 and *S. marcescens* C19 were used for comparative purposes.

### 4.6. Statistical Analysis

The phenotypic assays (biofilm formation, motility) were tested for statistical significance with ANOVA, followed by Tuckey post hoc test (*p* < 0.05), with Statistica 13.3 (StatSoft Polska, Krakow, Poland). The principal component analysis (PCA) was also performed with the use of Statistica 13.3.

## Figures and Tables

**Table 1:** Overview of the meat samples obtained from 5 markets in Gdansk, Poland, during 2020.

Meat Type	Packaging				Summary	
	Vacuum		Tray			
	Samples	%	Samples	%	Samples	%
turkey	33	38.4%	2	2.3%	35	40.7%
chicken	4	4.7%	13	15.1%	17	19.8%
pork	15	17.4%	4	4.7%	19	22.1%
beef	4	4.7%	11	12.8%	15	17.4%
SUM	56		30		86	

**Table 2:** Overview of the microbial abundance on different meat samples collected in Gdansk, Poland, 2020. The results are expressed as mean CFU/cm^2^ of the tested meat with standard deviation. Lactose utilization was tested on MacConkey media, while mannitol utilization was tested on MSA media. The ESBL strains were enumerated on ChromAgar ESBL media.

Samples			Lactose +	Lactose −	*E. coli* ESBL	KESC ESBL	Mannitol +	CT-R	
								Samples	%
Meat type	Turkey	35	16.6 ± 27.5	27.3 ± 32.3	0.4 ± 0.8	6 ± 17.1	14.9 ± 25.5	15	43%
	Chicken	17	27 ± 40	25 ± 43.5	0.9 ± 2.4	9 ± 24.6	13.9 ± 23.5	5	33%
	Pork	19	14.76 ± 29	15.2 ± 16.3	0.05 ± 0.2	1.9 ± 3.6	5.2 ± 7.2	4	24%
	Beef	15	13.4 ± 24.8	15.8 ± 23.8	0.06 ± 0.2	5.8 ± 15.5	3.8 ± 6	4	21%
Packaging	Vacuum	56	23.4 ± 27.3	27.5 ± 26.9	0.7 ± 0.5	3.9 ± 19.3	15.1 ± 13.6	16	57%
	Tray	30	17.4 ± 33.9	21.3 ± 30.1	0.3 ± 1.9	5.6 ± 11.2	10.5 ± 27.7	12	43%
Total		86	17.4 ± 29.8	21.3 ± 27.9	0.3 ± 1.2	5.6 ± 16.9	10.5 ± 20	28	nd

**Table 3:** Cephalosporin-resistant strains isolated from meat samples (*n* = 86) collected from markets in Gdansk. In a few cases, multiple strains were isolated from 1 sample.

Species	Meat Type				Packaging		Total	
	Turkey (*n* = 35)	Chicken (*n* = 17)	Pork (*n* = 19)	Beef (*n* = 15)	Vacuum (*n* = 56)	Tray (*n* = 30)	Isolates	%
*En. cloacae*	2	1	2	2	4	3	7	17
*E. coli*	7	1	0	0	7	1	8	19.5
*K. pneumoniae*	5	0	1	0	3	3	6	14.6
*S. fonticola*	6	4	3	3	9	7	16	39
*S. liquefaciens*	2	0	1	0	3	0	3	7.3
*S. marcescens*	0	0	0	1	0	1	1	2.4
Total	22	6	7	6	26	15	41	

## Data Availability

Data contained within the article and supplementary materials are available on request from the authors.

## Supplementary Materials

The following are available online at https://www.mdpi.com/article/10.3390/molecules27134151/s1, Figure S1: Swarming (A)**,** swimming (B) and biofilm formation (C) ability at room temperature (black) and at 37 °C (grey) of tested cephalosporin-resistant strains isolated from meat samples. The results are presented as average for each species with standard deviation (*S. fonticola n* = 16; *S. liquefaciens* *n* = 3; *S. marcescens* *n* = 1; *En. cloacae* *n* = 7; *E. coli* *n* = 8, *K. pneumoniae* *n* = 6). C49—*E. coli* ATCC 25922; C19—*S. marcescens* KPD102-BA. The experiment was performed twice with at least 3 replicates. Statistically different results between each species tested at different temperatures were marked with an asterisk as tested with ANOVA followed by Tuckey post hoc test at *p* < 0.05. Table S1. Summary of the collected samples from 5 supermarkets in Gdansk, Poland (2020). The bacterial contamination was assessed with shown growth media. The isolated strains divided into different species are listed for each of the collected sample. Table S2. Third-generation cephalosporine-resistant strains (*n* = 41) isolated from meat samples during 2020 in supermarkets in Gdansk, Poland. Table S3. The results of antibiotic resistance testing of each isolated cephalosporin-resistant strain (*n* = 41).

## Sample Availability

Samples of the compounds are not available from the authors.

## References

[1] (2018). The Third OIE Annual Report on the Use of Antimicrobial Agents Intended for Use in Animals.

[2] EUCAST. http://www.eucast.org/expert_rules_and_intrinsic_resistance/.

[3] ECDC/EFSA/EMA (2015). First Joint Report on the Integrated Analysis of the Consumption of Antimicrobial Agents and Occurrence of Antimicrobial Resistance in Bacteria from Humans and Food-Producing Animals. EFSA J..

[4] Dandachi I., Chabou S., Daoud Z., Rolain J.M. (2018). Prevalence and Emergence of Extended-Spectrum Cephalosporin-, Carbapenem- and Colistin-Resistant Gram Negative Bacteria of Animal Origin in the Mediterranean Basin. Front. Microbiol..

[5] Esteve-Palau E., Solande G., Sanchez F., Sorli L., Montero M., Guerri R., Villar J., Grau S., Horcajada J.P. (2015). Clinical and economic impact of urinary tract infections caused by ESBL-producing *Escherichia coli* requiring hospitalization: A matched cohort study. J. Infect..

[6] (2013). Centers for Disease Control and Prevention. Antibiotic Resistance Threats in the United States. https://www.cdc.gov/drugresistance/pdf/ar-threats-2013-508.pdf.

[7] Tanner W.D., VanDerslice J.A., Goel R.K., Leecaster M.K., Fisher M.A., Olstadt J., MGurley C.M., Morris A.G., Seely K.A., Chapman L. (2019). Multi-state study of *Enterobacteriaceae* harboring extended-spectrum beta-lactamase and carbapenemase genes in U.S. drinking water. Sci. Rep..

[8] Rybak B., Wawrzyniak N., Wolska L., Potrykus M. (2021). *Escherichia coli* and *Serratia fonticola* ESBLs as a potential source of antibiotics resistance dissemination in the Tricity water reservoirs. Biochim. Pol..

[9] De Oliveira D.M.P., Forde B.M., Kidd T.J., Harris P.N.A., Schembri M.A., Beatson S.A., Paterson D.L., Walker M.J. (2020). Antimicrobial resist ance in ESKAPE pathogens. Clin. Microbiol. Rev..

[10] World Health Organization (2014). Antimicrobial Resistance: Global Report on Surveillance. https://www.who.int/drugresistance/documents/surveillancereport/en/.

[11] World Health Organization (2017). WHO Publishes List of Bacteria for Which New Antibiotics are Urgently Needed. http://www.who.int/mediacentre/news/releases/2017/bacteria-antibiotics-needed/en/.

[12] Pfeifer Y., Cullik A., Witte W. (2010). Resistance to cephalosporins and carbapenems in Gram-negative bacterial pathogens. Int. J. Med. Microbiol..

[13] Bush K., Jacoby G.A. (2010). Updated functional classification of beta-lactamases. Antimicrob. Agents Chemother..

[14] Pulss S., Semmler T., Prenger-Berninghoff E., Bauerfeind R., Ewers C. (2017). First report of an *Escherichia coli* strain from swine carrying an OXA-181 carbapenemase and the colistin resistance determinant MCR-1. Int. J. Antimicrob. Agents.

[15] Mathers A.J., Peirano G., Pitout J.D. (2015). The role of epidemic resistance plasmids and international high-risk clones in the spread of multidrug-resistant *Enterobacteriaceae*. Clin. Microbiol. Rev..

[16] Bouchillon S.K., Badal R.E., Hoban D.J., Hawser S.P. (2013). Antimicrobial susceptibility of inpatient urinary tract isolates of gram negative bacilli in the United States: Results from the study for monitoring antimicrobial resistance trends (SMART) program: 2009–2011. Clin. Ther..

[17] Kelly A.M., Mathema B., Larson E.L. (2017). Carbapenem-resistant *Enterobacteriaceae* in the community: A scoping review. Int. J. Antimicrob. Agents.

[18] Cekanova L., Kolar M., Chroma M., Sauer P., Sedlackova M., Koukalova D. (2009). Prevalence of ESBL-positive bacteria in the community in the Czech Republic. Med. Sci. Monit..

[19] Reuland E.A., Overdevest I.T., Al Naiemi N., Kalpoe J.S., Rijnsburger M.C., Raadsen S.A., Ligtenberg-Burgman I., van der Zwaluw K.W., Heck M., Savelkoul P.H. (2013). Highprevalence of ESBL-producing *Enterobacteriaceae* carriage in Dutch community patients with gastrointestinal complaints. Clin. Microbiol. Infect..

[20] Bush K. (2010). Alarming β-lactamase-mediated resistance in multidrug-resistant *Enterobacteriaceae*. Curr. Opin. Microbiol..

[21] Uyanik T., Gülel G.T., Alişarli M. (2021). Characterization of extended-spectrum beta-lactamase-producing Enterobacterales from organic and conventional chicken meats. Lett. Appl. Microbiol..

[22] Tanimoto K., Nomura T., Hashimoto Y., Hirakawa H., Watanabe H., Tomita H. (2021). Isolation of *Serratia fonticola* producing FONA, a minor extended-spectrum β-lactamase (ESBL), from imported chicken meat in Japan. Jpn. J. Infect. Dis..

[23] Yang Y.Q., Li Y.X., Lei C.W., Zhang A.Y., Wang H.N. (2018). Novel plasmid-mediated colistin resistance gene mcr-7.1 in *Klebsiella pneumoniae*. J. Antimicrob. Chemother..

[24] Guo S., Aung K.T., Leekitcharoenphon P., Tay M.Y., Seow K.L., Zhong Y., Ching L., Møller Aarestrup F., Schlundt J. (2021). Prevalence and genomic analysis of ESBL-producing *Escherichia coli* in retail raw meats in Singapore. J. Antimicrob. Chemoth..

[25] Mezhoud H., Chantziaras I., Iguer-Ouada M., Moula N., Garmyn A., Martel A., Touati A., Smet A., Haesebrouck F., Boyen F. (2016). Presence of antimicrobial resistance in coliform bacteria from hatching broiler eggs with emphasis on ESBL/AmpC-producing bacteria. Avian Pathol..

[26] Badr H., Reda R.M., Hagag N.M., Kamel E., Elnomrosy S.M., Mansour A.I., Shahein M.A., Ali S.F., Ali H.R. (2022). Multidrug-Resistant and Genetic Characterization of Extended-Spectrum Beta-Lactamase-Producing *E. coli* Recovered from Chickens and Humans in Egypt. Animals.

[27] Zurfluh K., Nuesch-Inderbinen M., Morach M., Berner A.Z., Hachler H., Stephan R. (2015). Extended-spectrum-beta-lactamase-producing *Enterobacteriaceae* isolated from vegetables imported from the Dominican Republic, India, Thailand, and Vietnam. Appl. Environ. Microbiol..

[28] De Rooij M.M., Hoek G., Schmitt H., Janse I., Swart A., Maassen C.B., Schalk M., van Leeuwenhoeklaan A., Heederik D.J.J., Wouters I.M. (2019). Insights into Livestock-Related Microbial Concentrations in Air at Residential Level in a Livestock Dense Area. Environ. Sci. Technol..

[29] Beyrouthy R., Robin F., Lessene A., Lacombat I., Dortet L., Naas T., Ponties V., Bonnet R. (2017). MCR-1 and OXA-48 in vivo acquisition in KPC-producing *Escherichia coli* after colistin treatment. Antimicrob. Agents Chemother..

[30] McLellan J.E., Pitcher J.I., Ballard S.A., Grabsch E.A., Bell J.M., Barton M., Grayson M.L. (2018). Superbugs in the supermarket? Assessing the rate of contamination with thirdgeneration cephalosporin-resistant gram negative bacteria in fresh Australian pork and chicken. Antimicrob. Resist. Infect. Control..

[31] Müller A., Jansen W., Grabowski N.T., Monecke S., Ehricht R., Kehrenberg C. (2018). ESBL- and AmpC-producing *Escherichia coli* from legally and illegally imported meat: Characterization of isolates brought into the EU from third countries. Int. J. Food Microbiol..

[32] Palmeira J.D., Ferreira H., Madec J.Y., Haenni M. (2018). Draft genome of a ST443 mcr-1- and blaCTX-M-2-carrying *Escherichia coli* from cattle in Brazil. J. Glob. Antimicrob. Resist..

[33] Riley L.W. (2020). Extraintestinal foodborne pathogens. Annu. Rev. Food Sci. Technol..

[34] Cyoia P.S., Koga V.L., Nishio E.K., Houle S., Dozois C.M., de Brito K.C.T., de Brito B.G., Nakazato G. (2018). Kobayashi, R.K.T. Distribution of ExPEC virulence factors, blaCTX-M, fosA3, and mcr-1 in *Escherichia coli* isolated from commercialized chicken carcasses. Front. Microbiol..

[35] Dominguez J.E., Faccone D., Tijet N., Gomez S., Corso A., Fernandez-Miyakawa M.E., Melano R.G. (2019). Characterization of *Escherichia coli* carrying mcr-1-plasmids recovered from food animals from Argentina. Front. Cell Infect. Microbiol..

[36] Davis G.S., Waits K., Nordstrom L., Weaver B., Aziz M., Gauld L., Grande H., Bigler R., Horwinski J., Porter S. (2015). Intermingled *Klebsiella pneumoniae* populations between retail meats and human urinary tract infections. Clin. Infect. Dis..

[37] Hasman H., Hammerum A.M., Hansen F., Hendriksen R.S., Olesen B., Agerso Y., Zankari E., Leekitcharoenphon P., Stegger M., Kaas R.S. (2015). Detection of mcr-1 encoding plasmid-mediated colistin-resistant *Escherichia coli* isolates from human bloodstream infection and imported chicken meat, Denmark 2015. Eurosurveillance.

[38] Liu Y.Y., Wang Y., Walsh T.R., Yi L.X., Zhang R., Spencer J., Doi Y., Tian G., Dong B., Huang X. (2016). Emergence of plasmid-mediated colistin resistance mechanism MCR-1 in animals and human beings in China: A microbiological and molecular biological study. Lancet Infect. Dis..

[39] Köck R., Daniels-Haardt I., Becker K., Mellmann A., Friedrich A.W., Mevius D., Schwarz S., Jurke A. (2018). Carbapenem-resistant *Enterobacteriaceae* in wildlife, food-producing, and companion animals: A systematic review. Clin. Microbiol. Infect..

[40] Roer L., Hansen F., Stegger M., Sönksen U.W., Hasman H., Hammerum A.M. (2017). Novel mcr-3 variant, encoding mobile colistin resistance, in an ST131 *Escherichia coli* isolate from bloodstream infection, Denmark, 2014. Eurosurveillance.

[41] (2019). EMA/CVMP/CHMP/682198/2017. Committee for Medicinal Products for Veterinary Use (CVMP); Committee for Medicinal Products for Human Use (CHMP), Categorisation of Antibiotics in the European Union. www.ema.europa.eu.

[42] Clemente L., Manageiro V., Correia I., Amaro A., Albuquerque T., Themudo P., Ferreira E., Caniça M. (2019). Revealing mcr-1-positive ESBL-producing *Escherichia coli* strains among *Enterobacteriaceae* from food-producing animals (bovine, swine and poultry) and meat (bovine and swine), Portugal, 2010–2015. Int. J. Food Microbiol..

[43] Doi Y., Paterson D.L., Pascual E.A., López-Cerero L., Navarro M.D., Adams-Haduch J.M., Qureshi Z.A., Sidjabat H.E., Rodríguez-Baño J. (2009). Extended-spectrum and CMY-type β-lactamasep-producing *Escherichia coli* in clinical samples and retial meat from Pittsburgh, USA and Sevile, Spain. Clin. Microbiol. Infect..

[44] Randall L.P., Lodge M.P., Elviss N.C., Lemma F.L., Hopkins K.L., Teale C.J., Woodford N. (2017). Evaluation of meat, fruit and vegetables from retail stores in five United Kingdom regions as sources of extended-spectrum beta-lactamase (ESBL)-producing and carbapenem-resistant *Escherichia coli*. Int. J. Food Microbiol..

[45] Kola A., Kohler C., Pfeifer Y., Schwab F., Kuhn K., Schulz K., Balau V., Breitbach K., Bast A., Witte W. (2012). High prevalence of extended-spectrum-b-lactamase-producing *Enterobacteriaceae* in organic and conventional retail chicken meat, Germany. J. Antimicrob. Chemother..

[46] Leverstein-van Hall M.A., Dierikx C.M., Stuart J.C., Voets G.M., van den Munckhof M.P., van Essen-Zandbergen A., Platteel T., Fluit A.C., van de Sande-Bruinsma N., Scharinga J. (2011). Dutch patients, retail chicken meat and poultry share the same ESBL genes, plasmids and strains. Clin. Microbiol. Infect..

[47] Carattoli A., Villa L., Feudi C., Curcio L., Orsini S., Luppi A., Pezzotti G., Magistrali C.F. (2017). Novel plasmid-mediated colistin resistance mcr-4 gene in *Salmonella* and *Escherichia coli*, Italy 2013, Spain and Belgium, 2015 to 2016. Eurosurveillance.

[48] Borowiak M., Fischer J., Hammerl J.A., Hendriksen R.S., Szabo I., Malorny B. (2017). Identification of a novel transposon-associated phosphoethanolamine transferase gene, mcr-5, conferring colistin resistance in d-tartrate fermenting *Salmonella enterica* subsp. *enterica* serovar Paratyphi. B. J. Antimicrob. Chemother..

[49] Camposa C.B., Fennerb I., Wieseb N., Lensingb C., Christnera M., Rohdea H., Aepfelbachera M., Fennerb T., Hentschke M. (2014). Prevalence and genotypes of extended spectrum beta-lactamases in *Enterobacteriaceae* isolated from human stool and chicken meat in Hamburg, Germany. Int. J. Med. Microbiol..

[50] Casella T., Lelles Nogueira M.C., Saras E., Haenni M. (2017). High prevalence of ESBLs in retail chicken meat despite reduced use of antimicrobials in chicken production, France. Int. J. Food Microbiol..

[51] von Tippelskirch P., Gölz G., Projahn M., Daehre K., Friese A., Roesler U., Alter T., Orquera S. (2018). Prevalence and quantitative analysis of ESBL and AmpC beta-lactamase producing *Enterobacteriaceae* in broiler chicken during slaughter in Germany. Int. J. Food Microbiol..

[52] Dierikx C.M., van der Goot J.A., Smith H.E., Kant A., Mevius D.J. (2013). Presence of ESBL/AmpC-producing *Escherichia coli* in the broiler production pyramid: A descriptive study. PLoS ONE.

[53] Hering J., Fromke C., von Munchhausen C., Hartmann M., Schneider B., Friese A., Rosler U., Kreienbrock L., Hille K. (2016). Cefotaxime-resistant *Escherichia coli* in broiler farms-a cross-sectional investigation in Germany. Prev. Vet. Med..

[54] Schill F., Abdulmawjood A., Klein G., Reich F. (2017). Prevalence and characterization of extended-spectrum beta-lactamase (ESBL) and AmpC beta-lactamase producing *Enterobacteriaceae* in fresh pork meat at processing level in Germany. Int. J. Food Microbiol..

[55] Machado E., Coque T.M., Canton R., Sousa J.C., Peixe L. (2008). Antibiotic resistance integrons and extended-spectrum β-lactamases among *Enterobacteriaceae* isolates recovered from chickens and swine in Portugal. J. Antimicrob. Chemother..

[56] Geser N., Stephan R., Hachler H. (2012). Occurrence and characteristics of extended spectrum beta-lactamase (ESBL) producing *Enterobacteriaceae* in food producing animals, minced meat and raw milk. BMC Vet. Res..

[57] Depoorter P., Persoons D., Uyttendaele M., Butaye P., De Zutter L., Dierick K., Herman L., Imberechts H., Van Huffel X., Dewulf J. (2012). Assessment of human exposure to 3^rd^ generation cephalosporin resistant *E. coli* (CREC) through consumption of broiler meat in Belgium. Int. J. Food Microbiol..

[58] Tschudin-Sutter S., Frei R., Stephan R., Hachler H., Nogarth D., Widmer A.F. (2014). Extended-spectrum beta-lactamase (ESBL)-producing *Enterobacteriaceae*: A threat from the kitchen. Infect. Control. Hosp. Epidemiol..

[59] Diaz-Jimenez D., Garcia-Menino I., Fernandez J., Garcia V., Mora A. (2020). Chicken and turkey meat: Consumer exposure to multidrug-resistant *Enterobacteriaceae* including *mcr*-carriers, uropathogenic *E. coli* and highrisk lineages such as ST131. Int. J. Food Microbiol..

[60] Zowawi H.M., Harris P.N., Roberts M.J., Tambyah P.A., Schembri M.A., Pezzani M.D., Williamson D.A., Paterson D.L. (2015). The emerging threat of multidrug-resistant gram-negative bacteria in urology. Nat. Rev. Urol..

[61] (2020). National Agricultural Support Center; Analysis and Strategy Office. Polish Foreign Trade in Meat Products in 2019, Warsaw. www.kowr.gov.pl.

[62] Polkowska E. Wykorzystywanie Antybiotyków w Produkcji Zwierzęcej w Województwie Lubuskim, Najwyższa Izba Kontroli, Warszawa. www.nik.gov.pl.

[63] Krasucka D., Biernacki B., Szumiło J., Burmańczuk A. (2017). Monitoring zużycia leków przeciwdrobnoustrojowych u bydła, trzody chlewnej i koni w Polsce w latach 2014–2016 na podstawie Programu Wieloletniego. Życie Weter..

[64] Granato D., Santos J.S., Escher G.B., Ferreira B.L., Maggio R.M. (2018). Use of principal component analysis (PCA) and hierarchical cluster analysis (HCA) for multivariate association between bioactive compounds and functional properties in foods: A critical perspective. Trends Food Sci. Technol..

[65] OECD (2022). Meat Consumption.

[66] Central Statistical Office (GUS) (2020). Agriculture in 2019, Warsaw.

[67] Data Sheet on Poultry Production in Europe, Agridata. https://ec.europa.eu/info/sites/default/files/food-farming-fisheries/farming/documents/poultry-meat-dashboard_en.pdf.

[68] Data Sheet on Pigs Production in EUROPE, Agridata. https://agridata.ec.europa.eu/Reports/Pigmeat_Dashboard.pdf.

[69] Bergšpica I., Kaprou G., Alexa E.A., Prieto M., Alvarez-Ordóñez A. (2020). Extended spectrum β-lactamase (ESBL) producing *Escherichia coli* in pigs and pork meat in the European Union. Antibiotics.

[70] European Food Safety Authority, European Centre for Disease Prevention and Control (2020). The European Union Summary Report on Antimicrobial Resistance in zoonotic and indicator bacteria from humans, animals and food in 2017/2018. EFSA J..

[71] Melzer M., Petersen I. (2007). Mortality following bacteraemic infection caused by extended spectrum beta-lactamase (ESBL) producing *E. coli* compared to non-ESBL producing *E. coli*. J. Infect..

[72] MacKinnon M.C., McEwen S.A., Pearl D.L., Parfitt E.C., Pasquill K., Steele L., Laupland K.B. (2021). *Escherichia coli* bloodstream infections in the western interior of British Columbia, Canada: A population-based cohort study. Epidemiol. Infect..

[73] van den Bunt G., van Pelt W., Hidalgo L., Scharringa J., de Greeff S.C., Schürch A.C., Mughini-Gras L., Bonten M.J.M., Fluit A.C. (2019). Prevalence, risk factors and genetic characterisation of extended-spectrum beta-lactamase and carbapenemase-producing *Enterobacteriaceae* (ESBL-E and CPE): A community-based cross-sectional study, the Netherlands, 2014 to 2016. Eurosurveillance.

[74] Kaesbohrer A., Bakran-Lebl K., Irrgang A., Fischer J., Kämpf P., Schiffmann A., Werckenthin C., Busch M., Kreienbrock L., Hille K. (2019). Diversity in prevalence and characteristics of ESBL/pAmpC producing *E. coli* in food in Germany. Vet. Microbiol..

[75] Parvin M., Talukder S., Ali M., Chowdhury E.H., Rahman M., Islam M. (2020). Antimicrobial resistance pattern of *Escherichia coli* isolated from frozen chicken meat in Bangladesh. Pathogens.

[76] Kim Y.J., Moon J.S., Oh D.H., Chon J.W., Song B.R., Lim J.S., Heo E.J., Park H.J., Wee S.H., Sung K. (2018). Genotypic characterization of ESBL-producing *E. coli* from imported meat in South Korea. Food Res. Int..

[77] Rahman S.U., Ahmad S., Khan I. (2018). Incidence of ESBL-producing-*Escherichia coli* in poultry farm environment and retail poultry meat. Pak. Vet. J..

[78] Ojer-Usoz E., González D., Vitas A.I., Leiva J., García-Jalón I., Febles-Casquero A., de la Soledad Escolano M. (2013). Prevalence of extended-spectrum β-lactamase-producing *Enterobacteriaceae* in meat products sold in Navarra, Spain. Meat Sci..

[79] Huizinga P., Kluytmans-van den Bergh M., Rossen J.W., Willemsen I., Verhulst C., Savelkoul P.H., Friedrich A.W., García-Cobos S., Kluytmans J. (2019). Decreasing prevalence of contamination with extended-spectrum beta-lactamase-producing *Enterobacteriaceae* (ESBL-E) in retail chicken meat in the Netherlands. PLoS ONE.

[80] European Food Safety Authority, European Centre for Disease Prevention and Control (2021). The European Union Summary Report on Antimicrobial Resistance in zoonotic and indicator bacteria from humans, animals and food in 2018/2019. EFSA J..

[81] Evers E.G., Pielaat A., Smid J.H., van Duijkeren E., Vennemann F.B., Wijnands L.M., Chardon J.E. (2017). Comparative exposure assessment of ESBL-producing *Escherichia coli* through meat consumption. PLoS ONE.

[82] Özpınar H., Tekiner İ.H., Sarıcı B., Çakmak B., Gökalp F., Özadam A. (2017). Phenotypic Characterization of ESBL-and AmpC-Type Betalactamases in *Enterobacteriaceae* From Chicken Meat and Dairy Products. Ank. Üniversitesi Vet. Fakültesi Derg..

[83] Bello-López J.M., Cabrero-Martínez O.A., Ibáñez-Cervantes G., Hernández-Cortez C., Pelcastre-Rodríguez L.I., Gonzalez-Avila L.U., Castro-Escarpulli G. (2019). Horizontal Gene Transfer and Its Association with Antibiotic Resistance in the Genus *Aeromonas* spp.. Microorganisms.

[84] Giaouris E., Heir E., Hébraud M., Chorianopoulos N., Langsrud S., Møretrø T., Habimana O., Desvaux M., Renier S., Nychas G.J. (2014). Attachment and biofilm formation by foodborne bacteria in meat processing environments: Causes, implications, role of bacterial interactions and control by alternative novel methods. Meat Sci..

[85] Ehuwa O., Jaiswal A.K., Jaiswal S. (2021). *Salmonella*, Food Safety and Food Handling Practices. Foods.

[86] Jarlier V., Nicolas M.H., Fournier G., Philippon A. (1988). Extended broad-spectrum beta-lactamases conferring transferable resistance to newer beta-lactam agents in *Enterobacteriaceae*: Hospital prevalence and susceptibility patterns. Rev. Infect. Dis..

[87] Versalovic J., de Bruijn F.J., Lupski J.R. (1998). Repetitive Sequence-based PCR (rep-PCR) DNA Fingerprinting of Bacterial Genomes. Bacterial Genomes 437–454.

[88] Heras J., Domínguez C., Mata E., Pascual V., Lozano C., Torres C., Zarazaga M. (2015). GelJ—A tool for analyzing DNA fingerprint gel images. BMC Bioinform..

